# MPAgenomics: an R package for multi-patient analysis of genomic markers

**DOI:** 10.1186/s12859-014-0394-y

**Published:** 2014-12-14

**Authors:** Quentin Grimonprez, Alain Celisse, Samuel Blanck, Meyling Cheok, Martin Figeac, Guillemette Marot

**Affiliations:** MODAL team, Inria Lille-Nord Europe, Villeneuve-d’Ascq, France; Laboratoire Paul Painlevé, Université Lille 1, Villeneuve-d’Ascq, France; Inserm, U837, Team 3, Cancer Research Institute of Lille, Lille, France; Plate-forme de génomique fonctionnelle et structurale, IFR-114, Université Lille 2, Lille, France; EA 2694, Université Lille 2, Lille, France

**Keywords:** SNP arrays, Segmentation of genomic data, Marker selection, Multi-patient analysis, R package

## Abstract

**Background:**

Last generations of Single Nucleotide Polymorphism (SNP) arrays allow to study copy-number variations in addition to genotyping measures.

**Results:**

MPAgenomics, standing for multi-patient analysis (MPA) of genomic markers, is an R-package devoted to: (*i*) efficient segmentation and (*ii*) selection of genomic markers from multi-patient copy number and SNP data profiles. It provides wrappers from commonly used packages to streamline their repeated (sometimes difficult) manipulation, offering an easy-to-use pipeline for beginners in R.

The segmentation of successive multiple profiles (finding losses and gains) is performed with an automatic choice of parameters involved in the wrapped packages. Considering multiple profiles in the same time, MPAgenomics wraps efficient penalized regression methods to select relevant markers associated with a given outcome.

**Conclusions:**

MPAgenomics provides an easy tool to analyze data from SNP arrays in R. The R-package MPAgenomics is available on CRAN.

## Background

Genome-wide Single Nucleotide Polymorphism (SNP) arrays have been widely used over the past few years [1]. First generations were measuring only genetic variations of Single Nucleotide Polymorphisms, which are single base pair mutations at specific loci. Last generations (e.g. SNP5.0, SNP6.0) also include non-polymorphic probes in order to study copy-number variations along the genome in addition to genotyping measures. These arrays are especially used to study the impact of diseases, e.g. cancer, on the human genome.

Analyzing data from genome-wide SNP arrays within R requires several packages, e.g. aroma for normalization of Affymetrix®; SNP arrays [2,3], changepoint or cghseg for segmentation of copy number profiles [4], cghcall for labelling segments [5], and glmnet for penalized regressions [6]. Each package performs a specific task along the whole analysis but none of them is related to the others. Output formats of given packages are often not compatible with input formats required by the other, making their use tricky for beginners in R. One main contribution of the MPAgenomics R package is to aggregate these commonly used packages, providing wrappers to inter-relate them automatically.

At each step of the analysis a large amount of packages are available to perform normalization, segmentation or marker selection. A careful choice of only a few methods is required to provide an easy-to-use and efficient tool.

In this software article, we describe two different pipelines implemented in the R package MPAgenomics. Both of them perform the whole analysis from raw data to normalization, and then either successive segmented profiles, or a list of genomic markers selected from all available profiles.

## Implementation

MPAgenomics is implemented in R [7]. The package is divided in four main parts: data normalization, segmentation, calling and marker selection. Each part depends on different packages. MPAgenomics provides wrappers for some functions of these packages and facilitates the interaction between outputs and inputs of different functions. It remedies some problems with the wrapped packages such as confusing parameter names.

### Data normalization

The normalization process in MPAgenomics contains *technical biases correction* and *copy number and allele B fraction estimation*. Following [8], *allele B fraction* refers to the proportion of the total signal coming from allele B. Normalization methods are available for Affymetrix®; arrays (10K, 100K, 500K, GenomeWideSNP 5 & 6, and CytoScanHD). The estimation of the total copy number and allele B fraction is made by *CRMAv2* [9] originally implemented in the aroma packages. For studies with matched normal-tumor samples, a better estimation is suggested and implemented for the allele B fraction of the tumoral sample with the *TumorBoost* method [8].

The use of aroma packages is difficult for neophytes due to the strict folder architecture it requires and the documentation of the project which is mainly dedicated to experts able to criticize each method proposed and understand details of each procedure.

MPAgenomics provides documentation with a detailed example explaining how to quickly analyze data. The tutorial can be accessed in R by running the following commands:

library(MPAgenomics)

vignette(“MPAgenomics”)

More details on each step or wrapper are given to help advanced users to run each function separately.

Several features in the original aroma packages create new folders and files within the architecture. Matching files from different processes associated with a given sample can be tricky for neophytes. MPAgenomics implements a wrapper to build the folder architecture, check filenames automatically, process *CRMAv2* and *TumorBoost* normalization steps. Miscellaneous functions are also provided to ease some actions like signal extraction. Furthermore, different graphs such as the copy number profile can be saved in the working directory for further visualization.

The following steps (segmentation, calling and/or selection of genomic markers) are available in two settings. One is aroma-based and exploits the folder architecture and the files generated along the process. The second does not depend on aroma and allows advanced users to use their own normalized data.

### Segmentation

Although the use of manual annotations provides the best segmentation results [10], it appears essential for multi-patient analysis to avoid relying on them since they are time-consuming.

Therefore, following simulation results of [10], MPAgenomics wraps the CGHSEG [11,12] and PELT (Pruned Exact Linear Time) [13] segmentation methods which appeared to be those with the best overall performance.

PELT and CGHSEG methods fit a Gaussian maximum likelihood model but they differ in the way they choose the number of segments. CGHSEG requires the maximal number of segments as input. In MPAgenomics, the optimal number of segments is chosen according to a penalty $C\times K \times \left (2.5+\log \left (\frac {P}{K}\right)\right)$ with a profile of length *P*, *K* the number of segments and *C*>0 a parameter to choose [14]. This choice is performed using slope heuristics [15]. The PELT method returns a segmentation with a number of segments automatically chosen by the algorithm according to a penalty *Kρ* log(*P*) with *ρ*>0 a parameter to choose. The choice of the penalty parameter has been raised in [4]. MPAgenomics suggests an automatic sample-specific choice of *ρ* chromosome by chromosome (see package vignette for details on the method). In MPAgenomics, the two methods, CGHSEG with the slope heuristic and PELT with our calibration method, are proposed. By default, CGHSEG is used because it is quicker than PELT due to the multiple execution required by the *ρ* calibration method we propose.

The implemented segmentation methods are independently available for both copy number and allele B fraction profiles. In the case of allele B fraction segmentation, only heterozygous SNPs are kept. First, a naive genotype call [8] is performed on each normal sample in order to separate heterozygous SNPs from homozygous SNPs. Naive genotyping method assumes SNPs are bi-allelic and therefore is not recommended for tumor samples. Thus allele B fraction segmentation in MPAgenomics requires matched normal-tumor pairs. Then, following [16], the resulting signal is centered on 0.5 and symmetrized, which makes it similar to the usual copy number.

### Calling

From each segmented profile, the *CGHcall* method [17] is run to label every copy-number segment in terms of *loss*, *normal*, and *gain*.

*CGHcall* depends on a parameter, named *cellularity*, corresponding to the contamination of a sample with healthy cells. In MPAgenomics, this parameter can be modified by users, by default its value is 1 meaning that tumor samples are pure.

In the aroma-dependent function, segmentation and calling are performed with the same wrapper. The calling is run for each profile separately. Results are saved in text format in the working folder architecture.

### Selection of genomic markers

The goal is to select genomic markers (e.g. SNPs or CNV) associated with a given response from all patient profiles simultaneously. There is no need to perform segmentation and calling before the multi-patient analysis, marker selection is made over all copy-number profiles. However, segmentation can be performed before marker selection if wanted, in order to reduce the noise and the dimensionality of the problem.

Assuming *I* individuals and *P* potential markers, then for each individual *i*, *y*_*i*_ denotes the response and *x*_*i*,*p*_ the corresponding normalized value of copy number or allele B fraction signal at genomic position *p*.

Due to the huge number of markers (*P*>>*I*), MPAgenomics uses by default the *lasso* [18] regularization method to select very few ones. This method offers two advantages: (*i*) it selects only few variables, easing the interpretability of results, (*ii*) there exist some algorithms such as the lars [19] to solve quickly the *lasso* problem and support high-dimensional data.

The lasso regularization method consists in minimizing $g_{\lambda } : \beta \in \mathbb {R}^{P}\mapsto g_{\lambda }(\beta)$, where

$$\begin{aligned} g_{\lambda}(\beta) = \sum\limits_{i=1}^{I} \left(y_{i}-(X\beta)_{i}\right)^{2}+\lambda \sum\limits_{p=1}^{P} |\beta_{p}|~~~, \end{aligned} $$

with $(X\beta)_{i}=\sum _{p} x_{i,p}\beta _{p}$ and *λ*>0 controlling the number of non-zero coordinates of *β*. After minimization, non-zero coefficients *β*_*p*_ correspond to influential positions to predict the response.

MPAgenomics genomic marker selection drastically improves currently available packages in terms of computation time. With the linear regression model, it efficiently provides the exact solution by using the new R package HDPenReg, which is an optimized implementation of the *lars* algorithm [19] specially dedicated to a huge number of markers.

Since the theoretical grounding of Lasso when *P*>>*I* relies on a theoretical condition (see [20]) that cannot be easily checked in practice, the spike and slab algorithm [21,22] – a three steps algorithm performing filtering, estimation and variable selection – is also provided in MPAgenomics as an alternative.

Logistic regression is also available for binary responses. In this case, MPAgenomics wraps the glmnet package [6] in the whole process. Unlike HDPenReg it does not provide the exact solution but is computationally very efficient. With glmnet and HDPenReg, the regularization parameter *λ* is chosen by *k*-fold cross-validation [23]. The selected variables are the most relevant ones regarding the response.

## Discussion

MPAgenomics is mainly dedicated to beginners in SNP array analyses. It solves problems commonly encountered by neophytes such as interaction between different packages or specialized documentation dedicated to experts in the field. In addition, MPAgenomics suggests careful and automatic choices of crucial parameters at each part of the analysis.

To achieve simplicity of usage, MPAgenomics does not propose all options implemented in the wrapped packages, especially for normalization. However, outputs are generated in such a way that interaction between wrapped packages and MPAgenomics is facilitated. For example, the strict directory structure of aroma packages is built by MPAgenomics. Therefore, advanced users may directly use specific options of aroma to enhance their analysis without renormalizing data from scratch.

As specified in the data normalization section, segmentation, calling and marker selection steps can be performed without the use of aroma. This allows users to provide their own normalized data into matrices. This is useful for non-Affymetrix®; SNP arrays, CGH arrays or high-throughput sequencing data. For the latter, count data might need a variance-stabilizing transformation into Gaussian data before using current segmentation, calling and marker selection. For example, the Anscombe transform [24] can be used in addition to appropriate normalization specific to the used technology (target sequencing, whole-genome sequencing).

Currently, copy number and allele B fraction are segmented independently from each other. Research is ongoing to propose joint segmentation methods allowing to detect uniparental disomies, fragments which present a normal copy number but a loss of heterozygosity in the corresponding allele B fraction.

## Conclusions

MPAgenomics provides user-friendly wrappers for normalization and multi-patient analysis of high-throughput genomic data. It offers a guideline for beginners in copy-number variation analysis focusing on proven methods for their effectiveness. MPAgenomics also provides automatic choices of crucial parameters for segmentation and selection of markers.

Even though normalization is provided for Affymetrix®; arrays, other steps (segmentation, calling, and marker selection) can be applied to normalized data from other DNA arrays and next-generation sequencing data.

## Availability and requirements

**Project name:** MPAgenomics**Project home page:**http://cran.at.r-project.org/package=MPAgenomics**Operating system(s):** Platform independent **Programming language:** R**Other requirements:** none **License:** GNU GPL (>=2) **Any restrictions to use by non-academics:** None
